# Identification of exosomal microRNAs and related hub genes associated with imatinib resistance in chronic myeloid leukemia

**DOI:** 10.1007/s00210-024-03198-1

**Published:** 2024-06-25

**Authors:** Arzu Zeynep Karabay, Tulin Ozkan, Aynur Karadag Gurel, Asli Koc, Yalda Hekmatshoar, Asuman Sunguroglu, Fugen Aktan, Zeliha Buyukbingöl

**Affiliations:** 1 Department of Biochemistry, Faculty of Pharmacy, Ankara University, Ankara, Turkey; 2 Department of Medical Biology, Faculty of Medicine, Ankara University, Ankara, Turkey; 3 Department of Medical Biology, Faculty of Medicine, Usak University, Usak, Turkey; 4 Department of Medical Biology, Faculty of Medicine, Altinbas University, Istanbul, Turkey

**Keywords:** Imatinib, Leukemia, miRNA, Exosome, MDR1, Resistance

## Abstract

Chemotherapy resistance is a major obstacle in cancer therapy, and identifying novel druggable targets to reverse this phenomenon is essential. The exosome-mediated transmittance of drug resistance has been shown in various cancer models including ovarian and prostate cancer models. In this study, we aimed to investigate the role of exosomal miRNA transfer in chronic myeloid leukemia drug resistance. For this purpose, firstly exosomes were isolated from imatinib sensitive (K562S) and resistant (K562R) chronic myeloid leukemia (CML) cells and named as Sexo and Rexo, respectively. Then, miRNA microarray was used to compare miRNA profiles of K562S, K562R, Sexo, Rexo, and Rexo-treated K562S cells. According to our results, miR-125b-5p and miR-99a-5p exhibited increased expression in resistant cells, their exosomes, and Rexo-treated sensitive cells compared to their sensitive counterparts. On the other hand, miR-210-3p and miR-193b-3p were determined to be the two miRNAs which exhibited decreased expression profile in resistant cells and their exosomes compared to their sensitive counterparts. Gene targets, signaling pathways, and enrichment analysis were performed for these miRNAs by TargetScan, KEGG, and DAVID. Potential interactions between gene candidates at the protein level were analyzed via STRING and Cytoscape software. Our findings revealed *CCR5*, *GRK2*, *EDN1*, *ARRB1*, *P2RY2*, *LAMC2*, *PAK3*, *PAK4*, and *GIT2* as novel gene targets that may play roles in exosomal imatinib resistance transfer as well as *mTOR*, *STAT3*, *MCL1*, *LAMC1*, and *KRAS* which are already linked to imatinib resistance. *MDR1* mRNA exhibited higher expression in Rexo compared to Sexo as well as in K562S cells treated with Rexo compared to K562S cells which may suggest exosomal transfer of *MDR1* mRNA.

**Graphical Abstract:**

**Figure:**
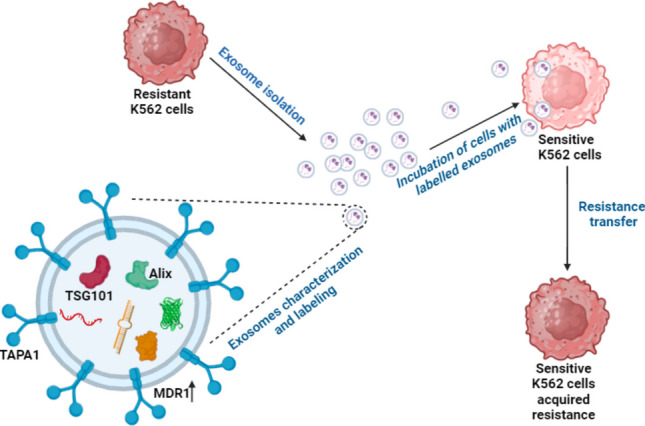
Figure is created with BioRender

**Supplementary Information:**

The online version contains supplementary material available at 10.1007/s00210-024-03198-1.

## Introduction

Exosomes are extracellular vesicles that are released into the extracellular space and transfer a wide variety of biocomponents such as DNAs, circRNAs, mRNAs, proteins, and miRNAs to other cells (Ratajczak et al. 2006; Simons and Raposo 2009; Lee et al. 2011; Fischer et al. 2016). Exosomes act as active signaling molecules between cancer cells and surrounding cells in the tumor microenvironment through the cargo they carry (Dai et al. 2020). miRNAs constitute an important component of the genetic cargo carried by exosomes. Recent findings have revealed that miRNAs released from exosomes during malignant transformation differ and changes in specific signaling pathways during carcinogenesis are mediated by the miRNAs released by cancer cells (Palma et al. 2012; Yi et al. 2020).

Chemotherapy resistance is one of the obstacles in cancer therapy, and exosomal miRNAs have been investigated as key molecules that confer drug resistance (Taghvimi et al. 2022). The transmittance of exosomal miRNAs has been reported in cisplatin resistance in gastric and breast cancers (Wang et al. 2019; Jing et al. 2022); gefitinib resistance in lung cancer (Jing et al. 2018); 5-FU resistance in colon cancer (Akao et al. 2014); and tamoxifen, adriamycin, and docetaxel resistance in breast cancer (Chen et al. 2014; Wei et al. 2014).

Another malignancy that is chemoresistant to therapy is chronic myeloid leukemia which is cancer of the bone marrow attributed to the constitutively active BCR-ABL oncogene. The success of the first-line therapy option BCR-ABL inhibitor imatinib has been opposed with drug resistance that occurs due to both intrinsic and extrinsic factors including ABL-kinase region mutations, BCR-ABL gene amplification/overexpression, clonal evolution, the presence of CML stem cells, the overexpression of MDR1 (multidrug resistance protein 1), and decreased drug bioavailability (Apperley 2007).

Recent studies have reported that exosomes and exosomal miRNAs may also play roles in the development of malignant behavior and imatinib resistance in CML (Zhu et al. 2014; Raimondo et al. 2015; Hrdinova et al. 2021). Exosomal transfer of *BCR-ABL1* mRNA from malignant cells to normal hematopoietic mononuclear cells was reported to induce leukemic malignancy (Zhu et al. 2014) whereas in another study exosomes derived from LAMA84 chronic myeloid leukemia cells were shown to induce the proliferation and survival of tumor cells both in vitro and in vivo (Raimondo et al. 2015). LAMA84 cell line-derived exosomes were also used in other studies which reported exosomal miR-126 as a mediator of communication between human umbilical vein endothelial cells (Taverna et al. 2014) and increased IL8 expression and tumor growth in CML cells by exosomes (Corrado et al. 2014). Another study analyzing the effects of CML derived exosomes on human umbilical vein endothelial cells revealed that the expression of ICAM-1 and VCAM-1 cell adhesion molecules and angiogenesis were increased (Taverna et al. 2012).

When we searched for studies investigating the role of exosomal miRNAs in CML and drug-resistant CML, miRNAs originating from K562 and K562 cell exosomes were analyzed in a study conducted in 2013, and the findings revealed the presence of differentially expressed miRNAs (Feng et al. 2013). On the other hand in another study, the expression of exosomal miRNAs from sensitive and resistant K562 cells was compared and miR-365, which was found as the most abundant exosomal miRNA in resistant cells was analyzed in sensitive cells treated with exosomes from resistant cells (Min et al. 2018). In this study, the difference in exosomal miRNA expression between resistant and sensitive CML cells was analyzed, and the role of the most abundant miRNA was studied. Unlike this study and in light of all related literature, we aimed to identify miRNAs involved in exosomal resistance transfer via a different approach by analyzing the miRNA profiles of imatinib-resistant and sensitive K562 cells, exosomes derived from them, and imatinib sensitive K562 cells treated with resistant cell exosomes via microarray. Our study also revealed novel interacting genes and proteins of identified significantly differently expressed miRNAs by computational methods.

## Materials and methods

### Chemicals

RPMI 1640 medium, fetal bovine serum (FBS), penicillin–streptomycin, and l-glutamine solutions were obtained from Gibco (Thermo-Fisher Scientific Inc., MO, USA). Primary antibodies for TSG-101, Alix, and TAPA were obtained from ATLAS; MDR1 and calnexin were obtained from Cell Signaling (Danvers, MA, USA); and secondary anti-rabbit Ig G-HRP and anti-mouse Ig G-HRP antibodies were obtained from Cell Signaling (Danvers, MA, USA). Imatinib was obtained from Santa Cruz Biotechnology (Santa Cruz, CA, USA). The loading buffer for the Western blot was obtained from New England Biolabs (Beverly, MA, USA). TRIzol was obtained from Invtirogen Biotechnology (Invitrogen, Carlsbad, USA). High Pure RNA kit, Transcriptor High Fidelity cDNA Synthesis Kit, and SYBR Green PCR Master Mix were obtained from Roche (Mannheim, Germany).

### Cell culture

K562S and K562R cells were cultured in RPMI-1640 medium supplemented with 10% FBS (fetal bovine serum), 100 U/ml penicillin, and 100 μg/ml streptomycin in an atmosphere of 95% air-5% CO_2_ at 37 °C. Exosome-free FBS was used in all studies. K562R cells were obtained from Prof. Carlo Gambacorti-Passerini, and their resistance to imatinib was increased up to 5 μM by incubating cells with increasing doses of imatinib. K562S cells were cultured in parallel with K562R cells without treatment with imatinib. In our past studies, we reported the genotypic and phenotypic characteristics of imatinib sensitive and resistant cells (Hekmatshoar et al. 2018, 2023). The administration of imatinib to resistant cells was discontinued two weeks before the start of the experiments to avoid any cytotoxic effects.

### Isolation and characterization of exosomes

**Fig. 1:**
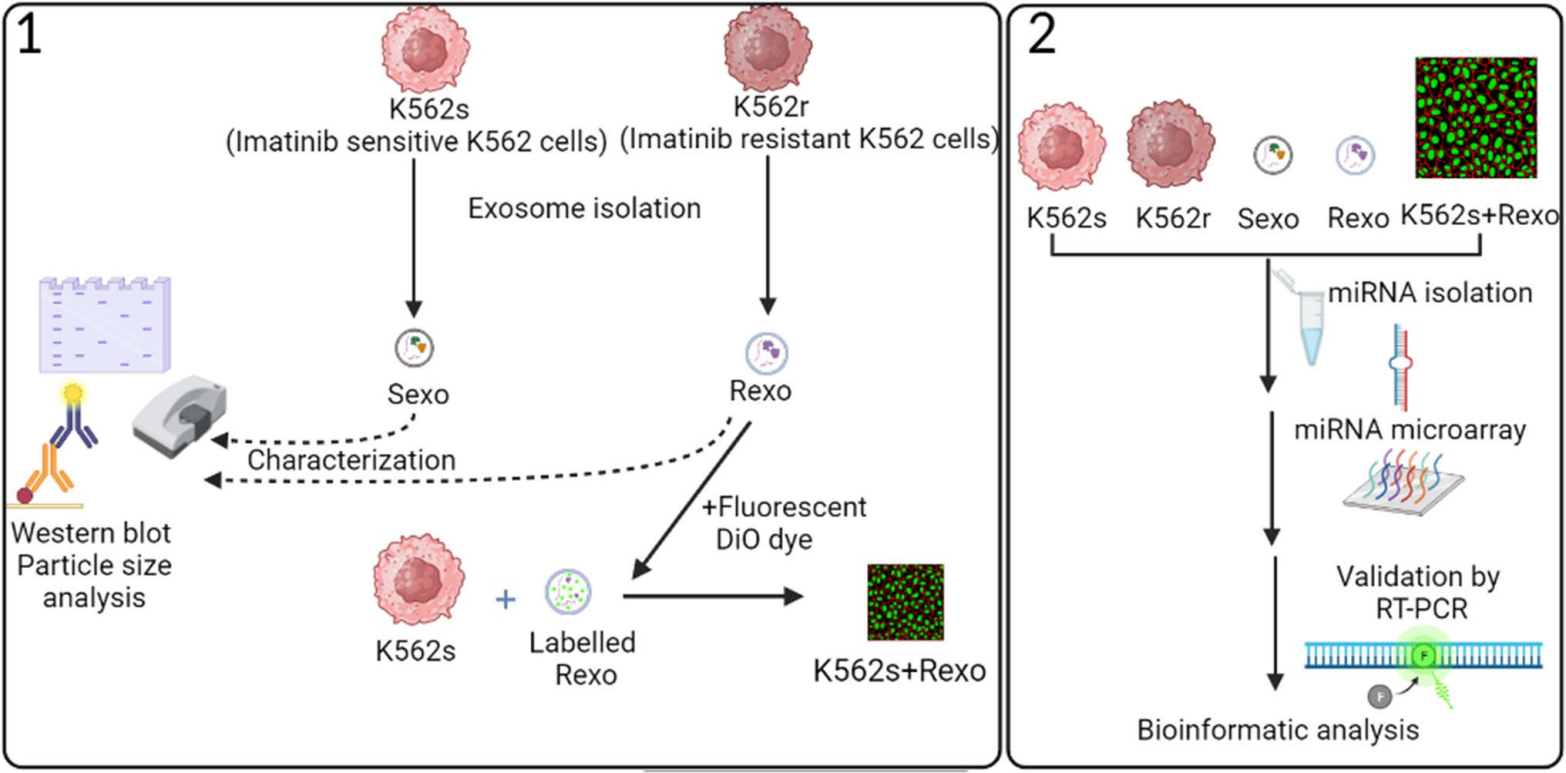
Schematic workflow of the methodology. **1** Exosome isolation, characterization and labeling. **2** Microrarray analysis of cellular and exosomal miRNAs and subsequent bioinformatic analysis are presented as a schematic workflow. Figure is created with BioRender

Exosomes were isolated from the medium of K562S and K562R cells (Fig. 1-1) according to the procedure of the miRCURY exosome isolation kit. Briefly, 10 ml volume of cell supernatant without cell debris was mixed with exosome precipitation solution. The tube was placed in the refrigerator at 4 °C and incubated for 1 h. After incubation, miRCURY/supernatant mixture was centrifuged at 3200 × g for 30 min. At the end of centrifugation, the exosomes appeared as white pellets at the bottom of the tube. Exosomal pellets were resuspended in sterile distilled water for particle analysis by Zeta Sizer Nano ZS (Malvern Instruments, Worcestershire, UK) at 25 °C (Sezgin-BayindirAntep and Yuksel, 2015). Protein content of exosomes was determined after the exosomes were lysed with lysis buffer. The proteins were analyzed using the steps mentioned below for characterization of the exosomes. Exosomes obtained from K562S and K562R cells were named Sexo and Rexo, respectively (Fig. 1-1).

Cellular and exosomal proteins were isolated using the lysis buffer (Active Motif Lysis Buffer AM1) of the protein isolation kit in accordance with the manufacturer’s protocol. The protein concentration was determined by the Bradford method using a spectrophotometer. Proteins were separated via electrophoresis on a 10% SDS polyacrylamide gel and transferred to polyvinylidene difluoride (PVDF) membranes. After 1.5 h of blocking with 5% milk powder in PBS (phosphate buffered saline), the membranes were incubated with anti-calnexin, anti-Tsg101, and anti-TAPA1 primary antibodies at 4 °C overnight and washed for 30 min with PBS-Tween solution. The next day, the membranes were incubated with an HRP-conjugated secondary antibody for 1 h at room temperature. After incubation and washing, the membranes were visualized under a CCD camera system using enhanced chemiluminescence (ECL) kit (Fig. 1-1).

### Labeling exosomes and incubation of cells with labeled exosomes

After isolation, the exosomes were incubated with Vybrant™ DiO dye (Fig. 1-1) which enables the follow-up of exosomes entering the cell via fluorescence microscopy and flow cytometry. Briefly, the following steps were followed. Exosomes were diluted with 100 µl of PBS. 5 μl/ml DiO dye was added to the pellet, and after mixing well, the mixture was stirred for 30 min and incubated at room temperature. After incubation, the cells were washed with 1 ml of PBS and after the washing step, the mixture was centrifuged at 3000 g for 30 min. The pellet was then diluted with 50 µl of PBS. Unstained exosomes were used as controls for flow cytometry analysis (Accuri C6) (Koc et al. 2018) and fluorescence microscopy.

### RNA extraction and microarray

**Fig. 2:**
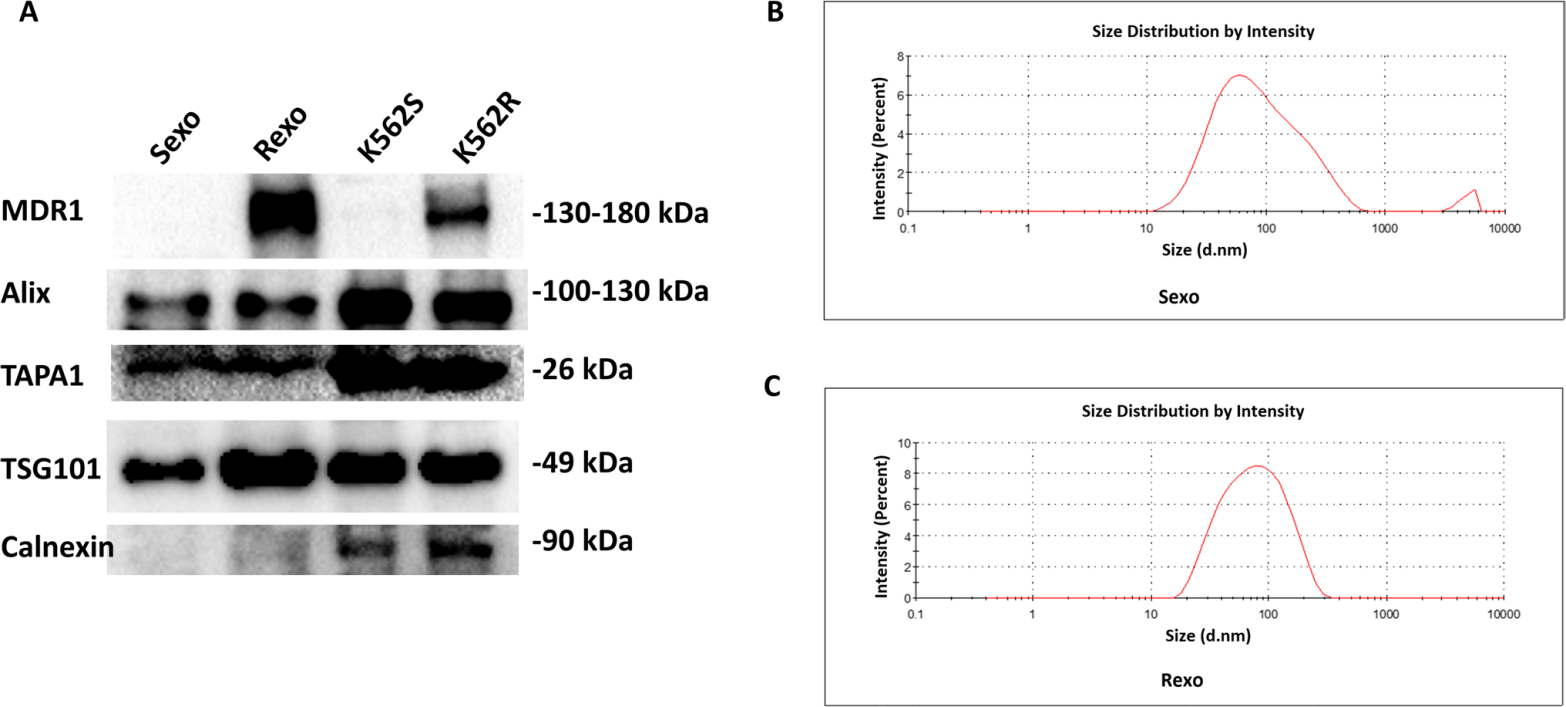
Characterization of exosomes via western blotting and particle size analysis. **A** Positive and negative exosomal markers in K562S, K562R cells, Sexo, and Rexo by western blot. Representative particle size analysis graphs of exosomes of **B** K562S and **C** K562R cells. *Y*-axes: signal intensity (%); *X*-axes: size of particles (nm)

RNA was isolated from different exosomes and cell preparations using total exosomal RNA, and a miRNA isolation kits in accordance with the manufacturer’s instructions (Fig. 1–2). The RNA was quantified spectrophotometrically and its quality was analyzed using an Agilent 2100 Bioanalyzer. To characterize the miRNA expression profiles of exosomes and exosome-treated cells and to detect differences in the expression profiles of the cells, a miRNA microarray was performed. In this sense, miRNAs from K562S, K562R, Sexo, Rexo, and K562S + Rexo were labeled using an exosomal RNA Flash Tag RNA labeling kit in accordance with the recommendations of the Affymetrix manufacturer. Next, cells were washed under standard conditions using an Affymetrix Fluidics Station 450 and a hybridization oven 640. Affymetrix Gene Array 3000 scanner was used to process the images, and Affymetrix miRNA QC Tool software was used to analyze the raw data files of the samples. The Affymetrix Gene Chip miRNA 3.0 Array included 19,724 probes and 1733 human adult miRNAs (Fig. 1–2). Pairwise comparisons of groups were made to exclude miRNAs with less than two fold changes.

### Bioinformatic analysis

#### Target gene analysis

TargetScan was used to identify miRNA targets. For selected miRNAs, target genes were also analyzed with KEGG (the Kyoto Encyclopedia of Genes and Genomes) database, and predominant biological pathways were determined for the selected miRNAs (Fig. 1–2).

#### Functional network establishment of miRNA target hub gene candidates

The functions of the overlapped differentially expressed (DE) miRNAs were identified by enrichment analysis on KEGG and GO pathways using the Database for Annotation, Visualization and Integrated Discovery v6.8 (DAVID) (https://david.ncifcrf.gov) which is a widely used well-grounded program that has different annotation tools to picture biological functions of proteins or genes. Gene ontology (GO) annotation includes three categories among which are biological process (BP), cellular component (CC), and molecular function (MF) that are used to demonstrate links between the gene and function. Pathway screening and significant functionality were determined by using the cut-off value which was set to *p* < 0.05.

#### miRNA-gene network construction and prognostic analysis

Three prominent programs were used to predict DE miRNA target genes among which were TargetScan (Lewis, Burge & Bartel, 2005) and miRDB (Wong & Wang, 2015) which possess known and validated miRNA interactions. The genes that were predicted by these two programs were chosen as DE miRNA targets and enabled the construction of a miRNA-gene network. MCODE, CytoHubba, and miRNA-gene networks were collectively used for identification of hub genes.

#### PPI network construction and app analysis

Predictions of interactions at the protein level between gene candidates were uncovered by using the Search Tool for the Retrieval of Interacting Genes database (version 10.0, http://string-db.org). Significance was adopted as a combined score of > 0.9 (high confidence score). Construction of PPI network was achieved by Cytoscape software (version 3.8.2, http://www.cytoscape.org/). PPI network modules were analyzed by another application, The Molecular Complex Detection (MCODE) app (Bandettini et al. 2012). Cut-off criteria was determined as MCODE scores ≥ 3 and the number of nodes > 5 with the default parameters (degree cut-off = 2, node score cut-off = 0.2, *k*-score = 2, and max. depth = 100). In addition, exploration of PPI network hub genes was made possible by CytoHubba, a Cytoscape plugin which has a user-friendly interface to discover crucial nodes in biological networks. Its computation uses 11 methods including MCC which successfully performs in the PPI network (Chin et al. 2014). Gene module pathway enrichment analysis was made by DAVID.

### Determination of specific gene expressions by real-time PCR

RNA extraction from cell pellets and exosomes was performed by using TRIzol (Invitrogen) as described by the manufacturer. The expression of the *MDR1* and *MCL1* mRNAs were determined. 1000 ng of RNA from each sample was converted to cDNA by Transcriptor High Fidelity cDNA Synthesis Kit (Roche) by following the protocol of the manufacturer. SYBR Green PCR Master Mix (Roche) was used for quantitative real-time RT–PCR which was performed on LC480 device. HPRT (hypoxanthine phosphoribosyl transferase) (Zand et al. 2024) was used as a housekeeping gene to detect mRNA expression in each sample. The cycling conditions were 10 min at 95 °C, 30 s at 95 °C, 30 s at the annealing temperature, and 30 s at 72 °C. PCR products were separated and visualized via 2% agarose gel electrophoresis (Karabay et al. 2024). Agarose gel and melt curve analysis were used to test whether the correct PCR products were amplified.

### Cell viability assays and IC50 calculations

The effect of imatinib on K562S, K562S + Rexo and K562R cells was determined with an MTT (methyl thiazole tetrazolium) assay. Briefly, cells were seeded and incubated with 0.037– 40 μM imatinib for 48 h. 20 μl of MTT (0.5 mg/ml) solution was added to each well for incubation at 37 °C for 4 h (Ozkan et al. 2021). After the incubation period, the formazan crystals were dissolved in a solubilizing agent, and absorbance at 550 nm was measured. Untreated cells served as controls, and their viability was set to 100%. The viability of treatment groups was calculated accordingly. The IC50 of imatinib was calculated with GraphPad Prism software.

### Statistical methods and calculations

Statistical analyses of the biological data which was in triplicate were conducted using GraphPad. A paired *t*-test was used to compare two datasets. The data is shown as the mean ± SD. Normality was verified using the Shapiro–Wilk or Kolmogorov–Smirnov test. Based on the normality of the data, comparisons between two groups were carried out using the *t*-test. Microarray data was analyzed as duplicate. Specific databases were used for bioinformatic analyses, and data with *P* < 0.05 were considered to be statistically significant.

## Results

### Characterization of exosomes by western blot and particle size analysis

Western blot results showed that negative exosomal marker calnexin was expressed in cells and but not in exosomes. The positive exosomal markers Alix, TSG101, and TAPA1 (CD81) were detected in the exosomal protein lysate which enabled confirmation of exosome isolation (Fig. 2A). In addition, it has been shown that Rexo, which originates from K562R cells, expresses very high levels of MDR1, an important mediator of drug resistance whereas K562S cells and their exosomes exhibit undetectable levels of this protein. These results supported the successful isolation of exosomes (Fig. 2A). Particle size analysis of the exosomes obtained from the Sexo and Rexo groups showed that they had exosomal characteristics (Fig. 2B, C).

### Labeling exosomes with DiO for cellular uptake

**Fig. 3:**
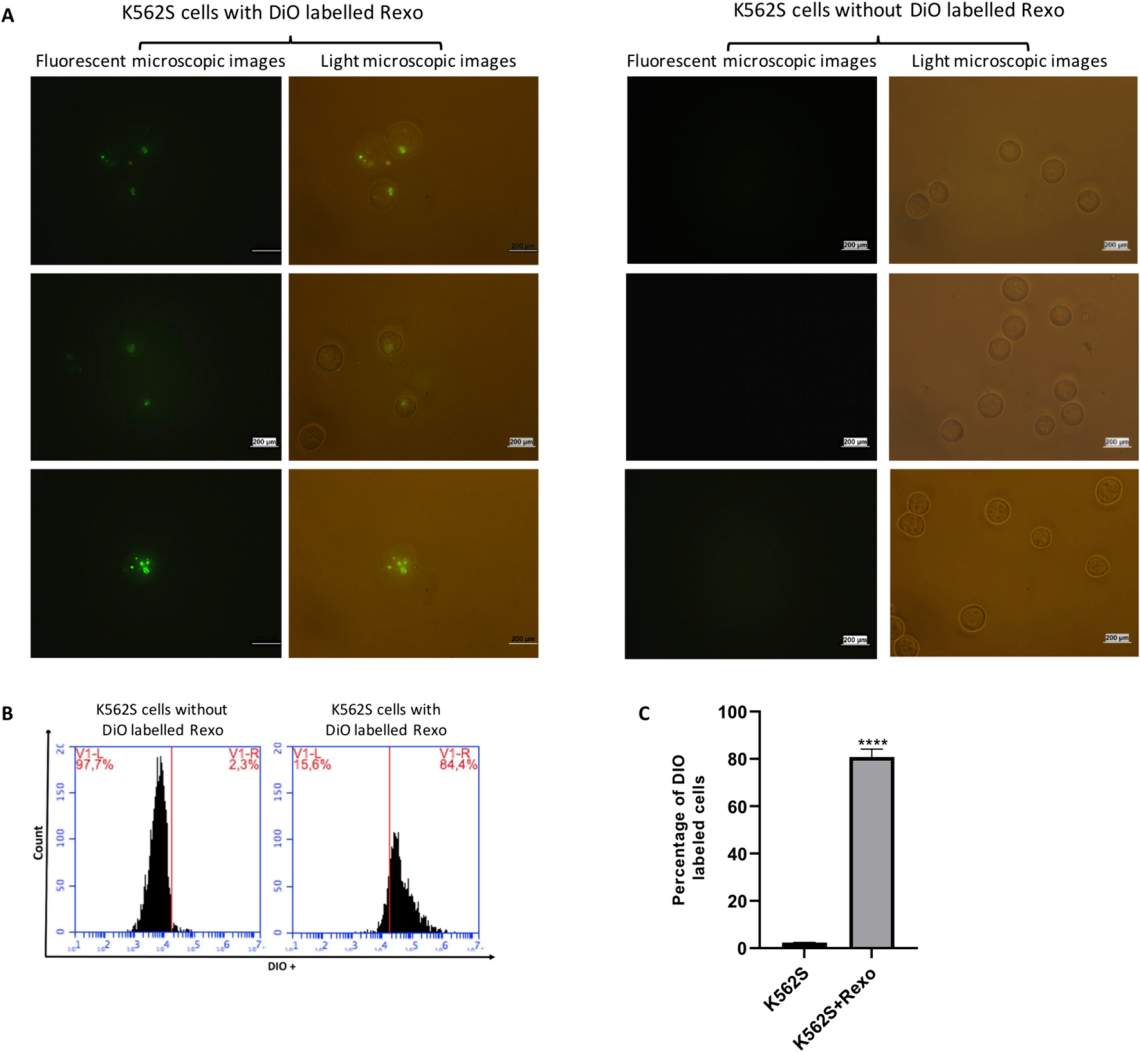
Microscopic and flow cytometric analysis of K562S and K562S + Rexo. **A** Fluorescence microscopic and light microscopic images of K562S cells incubated with DiO labeled Rexo (both white light and fluorescence were used to show the cells clearly with labeled exosomes) are shown in the left panel. Fluorescent and light microscopic images of K562S cells without labeled Rexo are shown on the right panel. The images show that the fluorescently labeled exosomes entered the cells. **B** Representative flow cytometry plots of K562S cells with or without DiO labeled Rexo. **C** Bar graph of the percentage of K562S cells with or without DiO labeled Rexo. Uptake of DiO labeled exosomes by K562S cells was confirmed by significant (****P ≤ 0.0001) increase in fluorescence intensity

Next, we confirmed the uptake of Rexo by K562S cells with fluorescence microscopy and flow cytometry. K562S cells incubated with DiO stained Rexo for 48 h were successfully visualized under a fluorescent microsope. The figure shows that the fluorescently labeled exosomes entered the cell (Fig. 3A). Uptake of DiO labeled exosomes by K562S cells was also confirmed by increased fluorescence intensity observed via flow cytometry (Fig. 3B).

### Microarray analysis of miRNAs in K562S, K562R cells, Sexo, Rexo, and K562S + Rexo

**Fig. 4:**
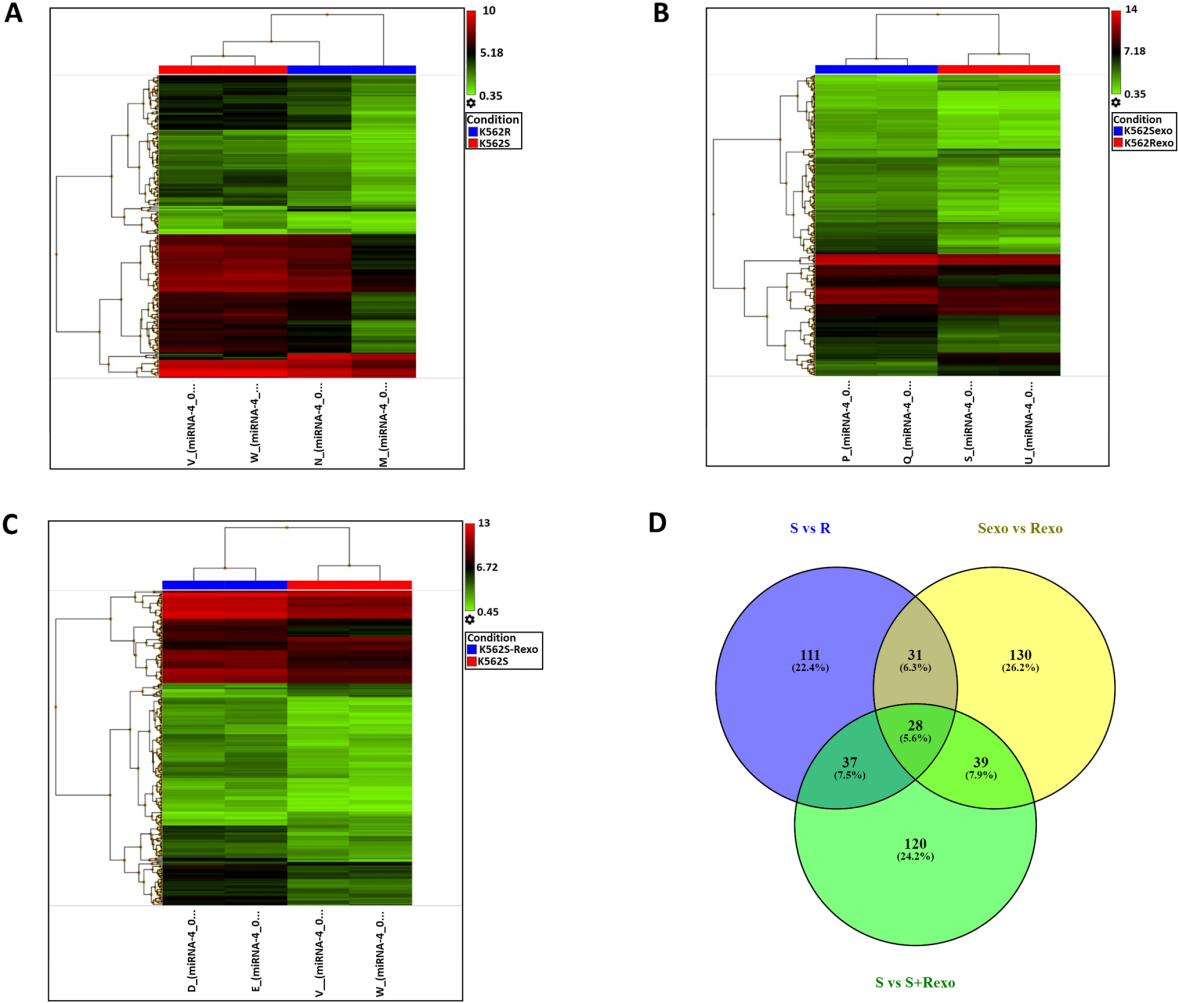
Heatmap plot and Venn diagram of cells. **A** Comparisons of the miRNA profiles of K562R and K562S. **B** Rexo and Sexo. **C** K562S and K562S + Rexo are illustrated by the hierarchical clustering heat map plots. **D** Venn diagram of common miRNAs is shown by intersecting differentially expressed miRNAs between S vs R, Sexo vs Rexo and K562S vs Rexo-treated K562S groups

We carried out microarray analysis of miRNAs in K562S, K562R, K562S + Rexo, Sexo, and Rexo. The data obtained from microarray analysis of miRNAs from K562S and K562R cells revealed that 207 miRNAs were differentially expressed between these two groups. Among the differentially expressed miRNAs, 11 miRNAs exhibited significantly increased expression in K562R cells compared with K562S cells, while the remaining 196 miRNAs exhibited significantly decreased expression in K562R cells (at least 2.0-fold change) (Fig. 4A).

Microarray analysis of Sexo and Rexo miRNAs revealed that 228 miRNAs were differentially expressed between these two groups. Among the differentially expressed miRNAs, 35 miRNAs exhibited increased expression in the Rexo group compared to the Sexo group, while the remaining 193 miRNAs exhibited decreased expression in the Rexo group. A comparison of the miRNA profiles between Rexo and Sexo is also illustrated by the hierarchical clustering plot (Fig. 4B).

A comparison of the miRNA profiles between Rexo-treated K562S cells and K562S cells revealed that 224 miRNAs were differentially expressed between these two groups. Among the differentially expressed miRNAs, 33 miRNAs exhibited increased expression in K562S compared to K562S + Rexo, while the remaining 191 miRNAs exhibited decreased expression in K562S compared to K562S + Rexo. A comparison of the miRNA profiles of K562S and K562S + Rexo is also shown by hierarchical clustering plot (Fig. 4C). A summary of differentially and commonly expressed miRNAs in all groups is also shown with a Venn diagram (Fig. 4D).

When the three groups of differentially expressed miRNAs, K562R vs K562S, Sexo vs Rexo, and K562S vs K562S + Rexo were intersected, 28 mRNAs were determined, and the results are given in Table 1 with fold change values.

**Table 1:**
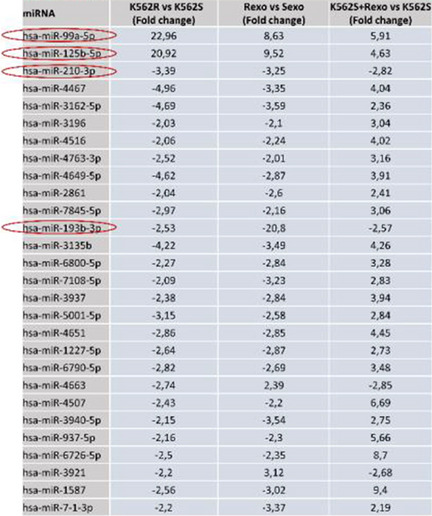
List of 28 mi RNAs with expression fold changes found in the intersection set in the Venn diagram

**Fig. 5:**
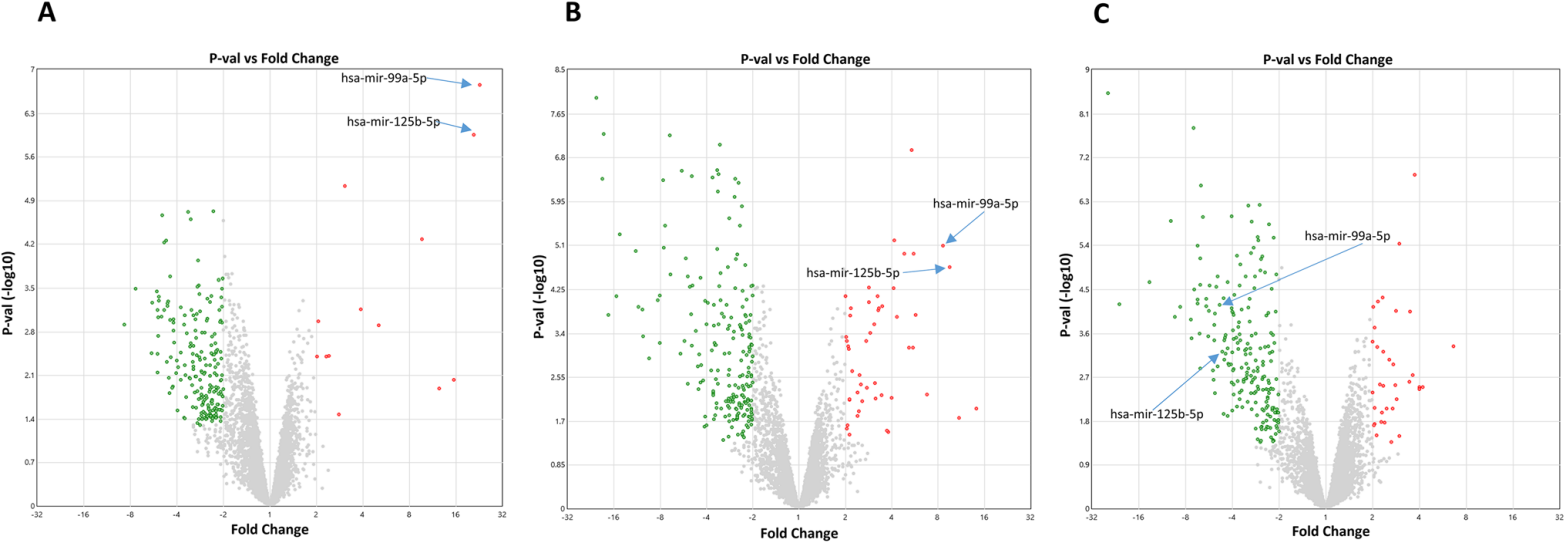
Volcano plots of differentially expressed miRNAs in the groups.** A** Sexo vs Rexo (the miRNAs in the Sexo and Rexo groups are depicted as green and red dots respectively). **B** K562S vs K562R (miRNAs of the K562S and K562R groups are depicted as green and red dots respectively). **C** K562S + Rexo vs K562S (miRNAs of K562S + Rexo and K562S groups are depicted as green and red dots, respectively). Two upregulated miRNAs, miR-99a-5p and miR-125b-5p, are representatively shown with blue arrows in each plot

Among these 28 miRNAs, miR-99a-5p and miR-125b-5p were found as the most abundantly expressed two miRNAs in resistant cells compared to sensitive cells with 22.96 and 20.92 fold expression changes, respectively. When we analyzed the expression of these two miRNAs in other groups, we also found that they exhibited higher expression in Rexo than in Sexo and in K562S + Rexo than in K562S cells. The expression levels of miR-99a-5p and miR-125b-5p were found as 8.63-fold and 9.52-fold higher in Rexo than Sexo and 5.91-fold and 4.63-fold higher in K562S + Rexo compared to K562S, respectively. Besides these two miRNAs with increased expression, miR-210-3p and miR-193b-3p were the only two miRNAs with decreased expression in the groups expressed above. Volcano plots of all of the miRNAs including miR-99a-5p, miR-125b-5p, miR-210-3p, and miR-193b-3p can also be seen as volcano plot (Fig. 5). The expression of miR-99a-5p and miR-125b-5p is representatively shown in the plot (Fig. 5).

### Functional enrichment analysis of differentially expressed genes (DEGs)

When the three groups were compared, the miRNAs whose expression increased or decreased in common in each group were selected with Venn diagram (Fig. 4D). According to the analysis results, the expression of two miRNAs miR-99a-5p and miR-125b-5p increased in common in all three groups, while the expression of two miRNAs miR-210-3p and miR-193b-3p decreased in common in all three groups. The target genes of these 4 selected miRNAs were predicted by the mirDB program. Based on these estimations, a total of 972 target genes were found for miRNAs with increased expression and 417 target genes were found for miRNAs with decreased expression.

The potential target genes of these 4 miRNAs, which are thought to be responsible for drug resistance transmission, were subjected to GO and KEGG pathway analyses, and Cytoscape software was used to construct a possible functional protein–protein interaction (PPI) network.

**Fig. 6:**
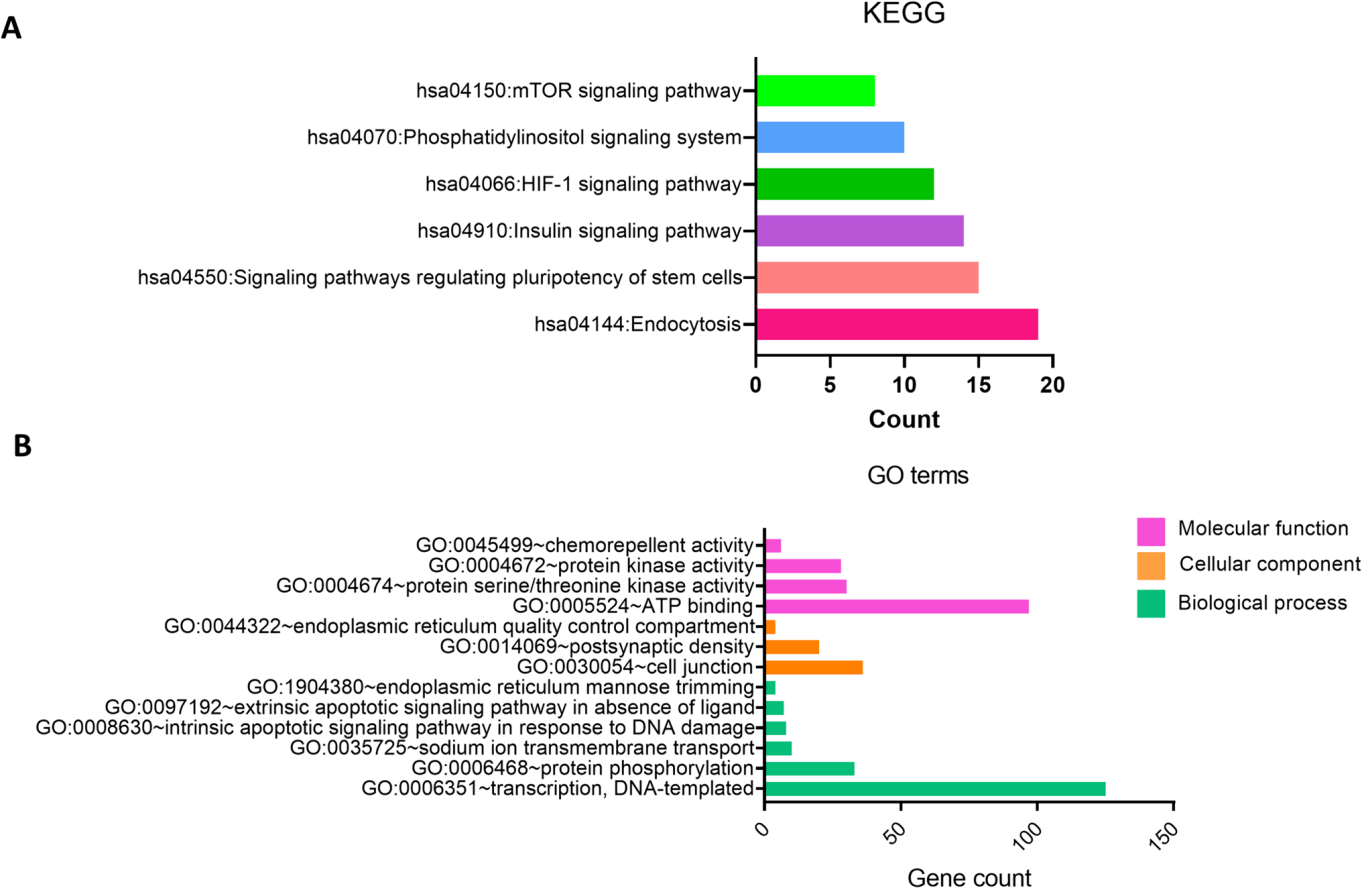
KEGG and GO enrichment analysis of highly expressed miRNAs. **A** KEGG pathway analysis of the 2 miRNAs with increased expression (miR-99a-5p, miR-125b-5p). The P significance values of KEGG pathway analyses were 0.0388, 0.0066, 0.0141, 0.0057, 0.0433, and 0.0195, respectively, for pathways between hsa04144:Endocytosis and hsa04150:mTOR signaling pathway. **B** GO analysis of the 2 miRNAs with increased expression (miR-99a-5p, miR-125b-5p). The corrected *p* value of less than 0.05 revealed the pathways in which the genes were most enriched. *P* significance values of GO terms were 0.0031, 0.0340, 0.0105, 0.0084, 0.0062, 0.0115, 0.0059, 0.0016, 0.0227, 0.0064, 0.0141, 0.0233, and 0.0100, respectively, for pathways between BP GO:0006351 ~ transcription, DNA-templated and MF GO:0045499 ~ chemorepellent activity

It has been found that miRNAs with increased expression play a role in a total of 7 pathways, among which is the MAPK signaling pathway with 5 genes for miR-99a-5p and 23 genes for miR-125b-5p. In addition to the MAPK signaling pathway, endocytosis (19 genes), signaling pathways regulating the pluripotency of stem cells (15 genes), HIF-1 signaling pathway (12 genes), and the phosphatidylinositol signaling system (10 genes) were found as interacting pathways (Fig. 6A).

**Fig. 7:**
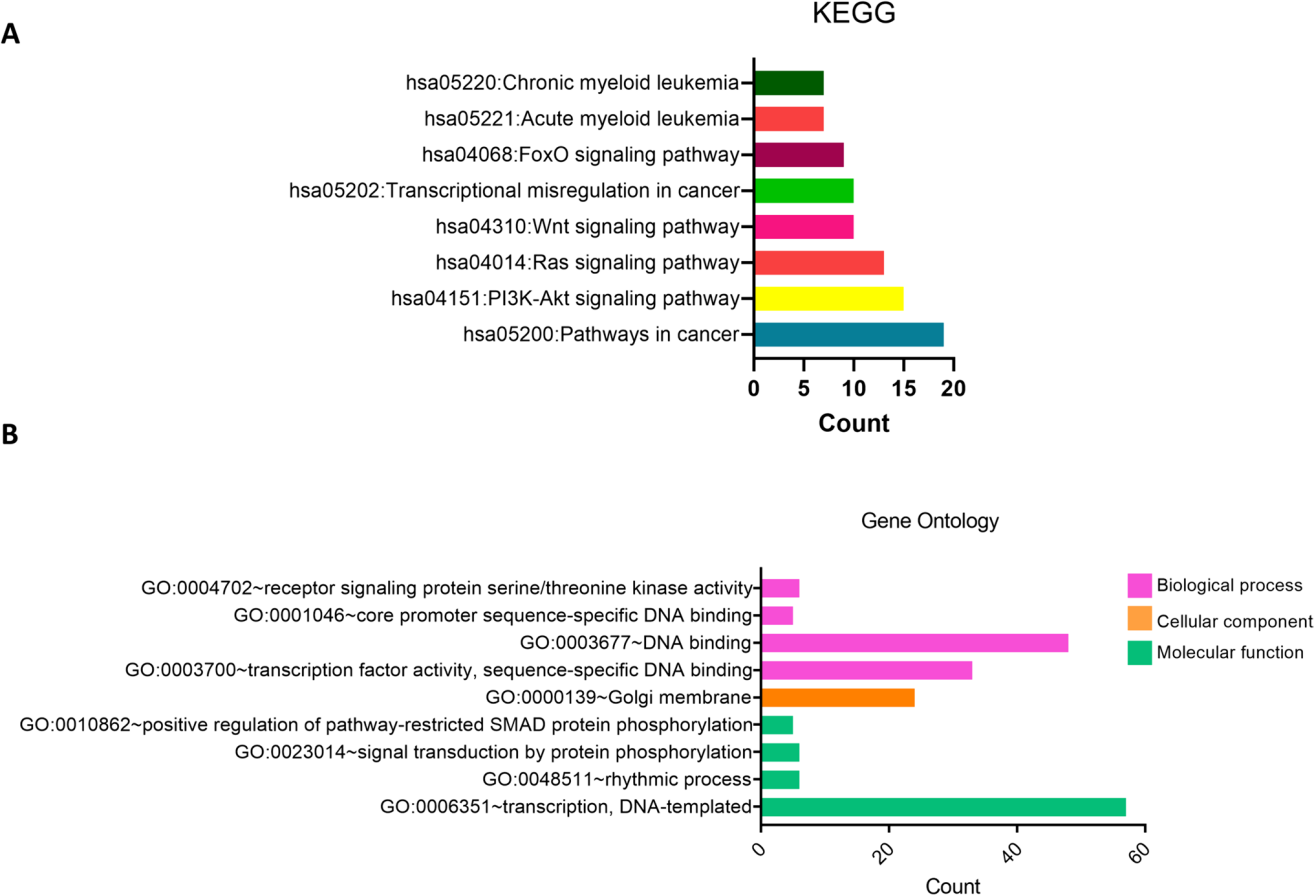
KEGG and GO enrichment analysis of lowly expressed miRNAs. **A** KEGG pathway analysis of the 2 miRNAs with decreased expression (miR-210-3p, miR-193b-3p). *P* significance values of KEGG pathway analyses were 0.0049, 0.0323, 0.0068, 0.0051, 0.0167, 0.0135, 0.0019, and 0.0067, respectively, for pathways between hsa05200:Pathways in cancer and hsa05220:Chronic myeloid leukemia. **B** GO analysis of the 2 miRNAs with decreased expression (miR-210-3p, miR-193b-3p). The corrected *p* value of less than 0.05 revealed the pathways in which the genes were most enriched. P significance values of GO terms were 0.0248, 0.0066, 0.0027, 0.0212, 0.0034, 0.0115, 0.0423, 0.0140, and 0.0058, respectively, for pathways between BP GO:0006351 ~ transcription, DNA-templated, and MF GO:0004702 ~ receptor signaling protein serine/threonine kinase activity

According to the pathway analyzes of the target genes of miR-210-3p and miR-193b-3p whose expression was decreased, the pathway in which the genes were most enriched was pathways in cancer (19 genes). The other pathways in which the genes were enriched are PI3K-Akt signaling pathway (15 genes), Ras signaling pathway (13), Wnt signaling pathway (10 genes), transcriptional misregulation in cancer (10 genes), ErbB signaling pathway (9 genes), FoxO signaling pathway (9 genes), acute myeloid leukemia (7 genes), and chronic myeloid leukemia (7 genes) (Fig. 7A).

### GO enrichment analysis

GO enrichment analysis, including biological process, molecular function, and cellular component, was performed with DAVID software to comprehensively investigate the biological functions of the target genes.

Target genes of the miRNAs with increased expression exhibited increased enrichment in the analysis of the biological processes especially transcription, DNA-templated (125 genes) and protein phosphorylation (33 genes) in BP, cell junction (36 genes), and postsynaptic density (20 genes) in CC. In MF, it was found to be enriched in ATP binding (97 genes) and protein serine/threonine kinase activity (30 genes). Other enriching processes are shown in Fig. 6B.

The target genes of the miRNAs with decreased expression were enriched in the transcription, DNA-templated (57 genes) in the BP category, the Golgi membrane (24 genes) in the CC category and DNA binding (48 genes), transcription factor activity, and sequence-specific DNA binding (35 genes) in the MF category (Fig. 7B).

### Screen modules and the top 10 hub genes of the PPI network via Cytoscape software

To evaluate the PPI information, we analyzed the target genes of the selected miRNAs via the use of the STRING database. According to the profile obtained from the STRING tool, the PPI network of target genes of miRNAs with increased expression contained 956 nodes and 274 edges, and the PPI enrichment *p* value was 0.00151. The highest number of modules was selected using the MCODE plugin, as the PPI network contains a large number of nodes and interactions.

Next, the modules of the PPI network were scanned by the Cytoscape software (version 3.7.1) plugin Molecular Complex Detection (MCODE) with the following default parameters: degree cut-off = 2, node score cut-off = 0.2, *k*-score = 2, and max. depth = 100. In this study, criteria of 11 modules were determined with MCODE scores ≥ 3 nodes (Table 2). The PPI network of target genes of increased miRNAs contained 414 nodes and 117 edges, and the PPI enrichment *p *value is 0.0092.

**Table 2:** Determination of criteria of 11 modules with MCODE (molecular complex detection) scores ≥ 3 nodes

Target hub genes of upregulated miRNAs				
Cluster	Score	Nodes	Edges	Node Ids
1	5	5	10	CCR5, ARRB1, GRK2, P2RY2, EDN1
2	4	4	6	PPP3CA, PPP1R9B, PPP2R5C, PPP2CA
3	4	4	6	LPCAT4, LCLAT1, MBOAT2, CDS2
4	3	3	3	UBE2W, UBE2R2, UBE2G1
5	3	3	3	PIP4K2B, PIK3R5, PI4K2B
6	3	7	9	TRAF6, UBE2L3, BMPR2, SMURF1, ITCH, BMPR1B, CER1
7	3	3	3	RAP1A, RAP1B, GAB2
8	3	3	3	ENPP1, UCK2, ENTPD1
9	3	7	9	RYBP, SUV39H1, E2F3, E2F2, PCGF6, BAP1, CBX7
10	3	3	3	MED20, CCNC, CDK19
11	3	3	3	DICER1, PRKRA, AGO2
Target hub genes of downregulated miRNAs				
Cluster	Score	Nodes	Edges	Node Ids
1	5	5	10	LAMC2, LAMC1, PAK3, PAK4, GIT2
2	3	3	3	AP2M1, KCNQ5, CLTC
3	3	3	3	ACVR1B, INHBB, ACVR2A

**Fig. 8:**
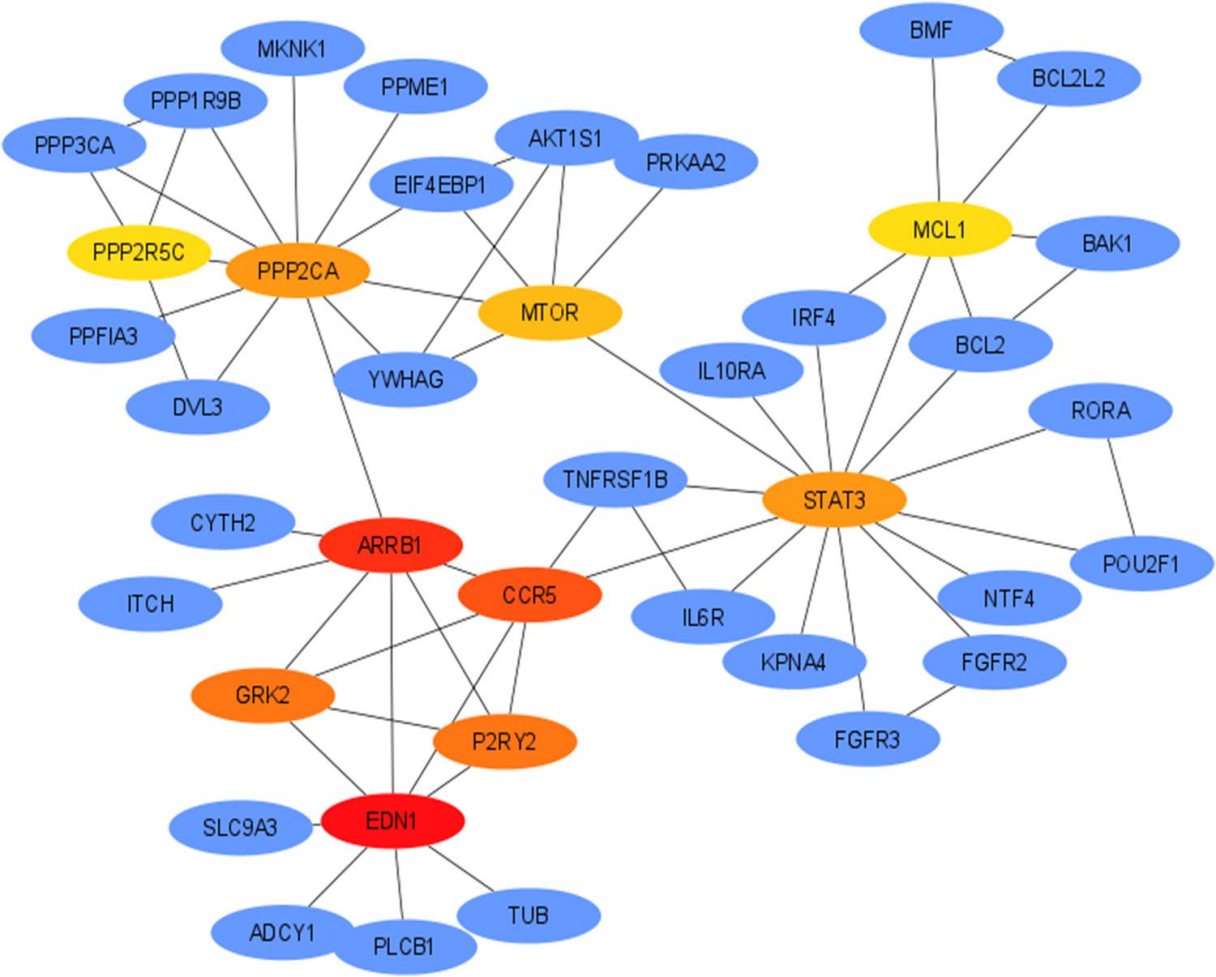
Protein–protein interaction (PPI) analysis and target PPI hub gene module of miRNAs with increased expression. The first 10 genes of the MMC method were chosen using CytoHubba plugin. A more forward ranking is represented by a redder color

Based on the highest degree of linkage, the top 10 genes were selected as target hub genes using the Cytoscape plugin CytoHubba. The ten genes with the highest ranking scores (*CCR5*, *GRK2*, *EDN1*, *ARRB1*, *P2RY2*, *PPP2CA*, *PPP2R5C*, *MTOR*, *STAT3*, and *MCL1*) stood out as target hub genes of miRNAs whose expression increased by applying CytoHubba add-on. These 10 genes (Fig. 8) are involved in chemokine signaling pathway, HIF-1 signaling pathway, PI3K-Akt signaling pathway, AMPK signaling pathway, microRNAs in cancer, and endocytosis pathways.

**Fig. 9:**
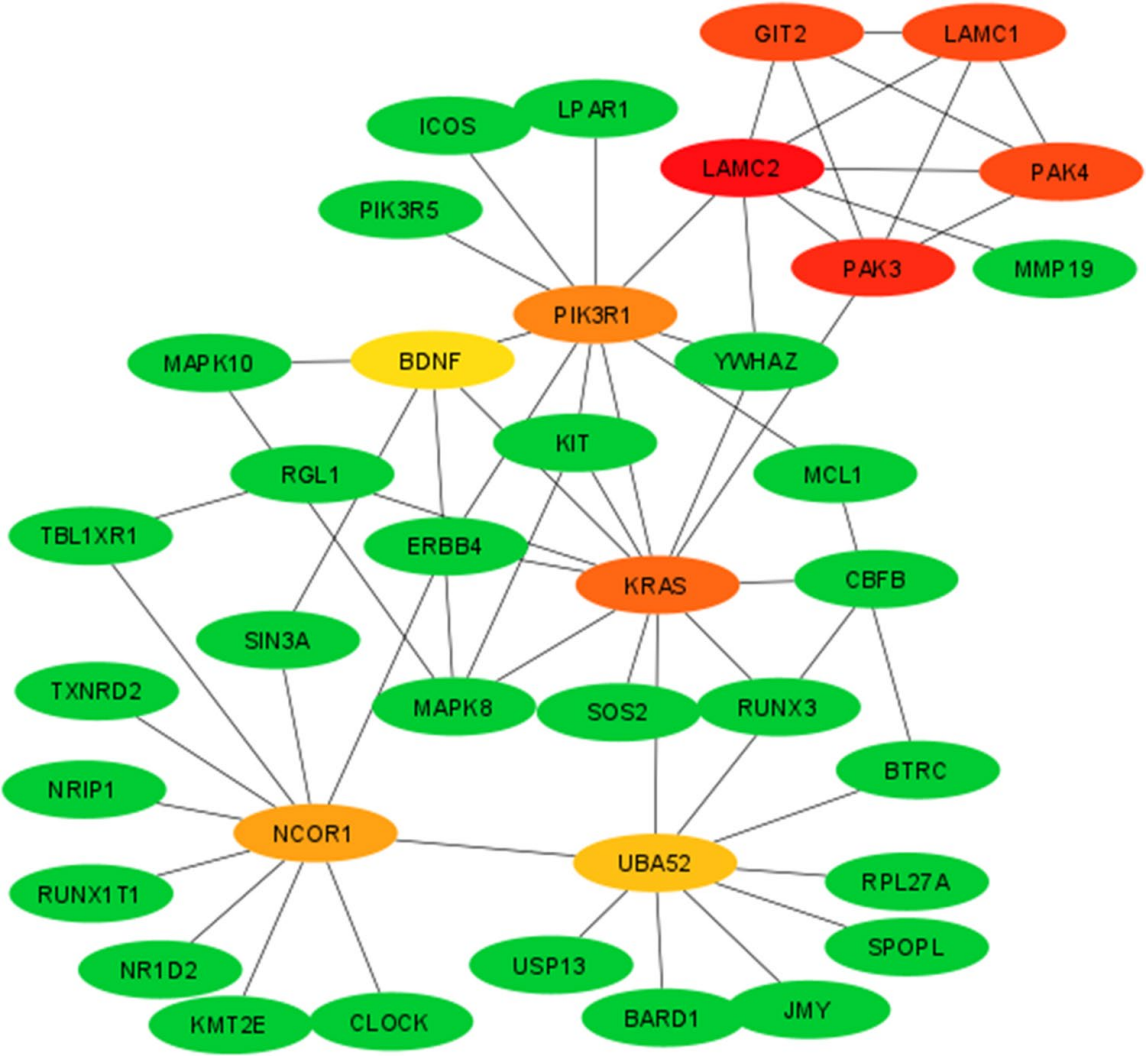
Protein–protein interaction (PPI) analysis and target PPI hub gene module of miRNAs with decreased expression. The first 10 genes of the MMC method were chosen using CytoHubba plugin. The more forward ranking is represented by a redder color

Based on the highest degree of association in target hub genes of miRNAs with decreased expression, the top 10 genes were found to be *LAMC2*, *PAK3*, *PAK4*, *LAMC1*, *GIT2*, *KRAS*, *PIK3R1*, *UBA52*, *NCOR1*, and *BDNF* (Fig. 9). The target hub genes of the miRNAs whose expression decreased were involved in the focal adhesion, ErbB signaling pathway, regulation of actin cytoskeleton, Ras signaling pathway, PI3K-Akt signaling pathway, and pathways in cancer pathways.

### Verification of miRNAs by identifying the expression of their target genes via RT-PCR

**Fig. 10:**
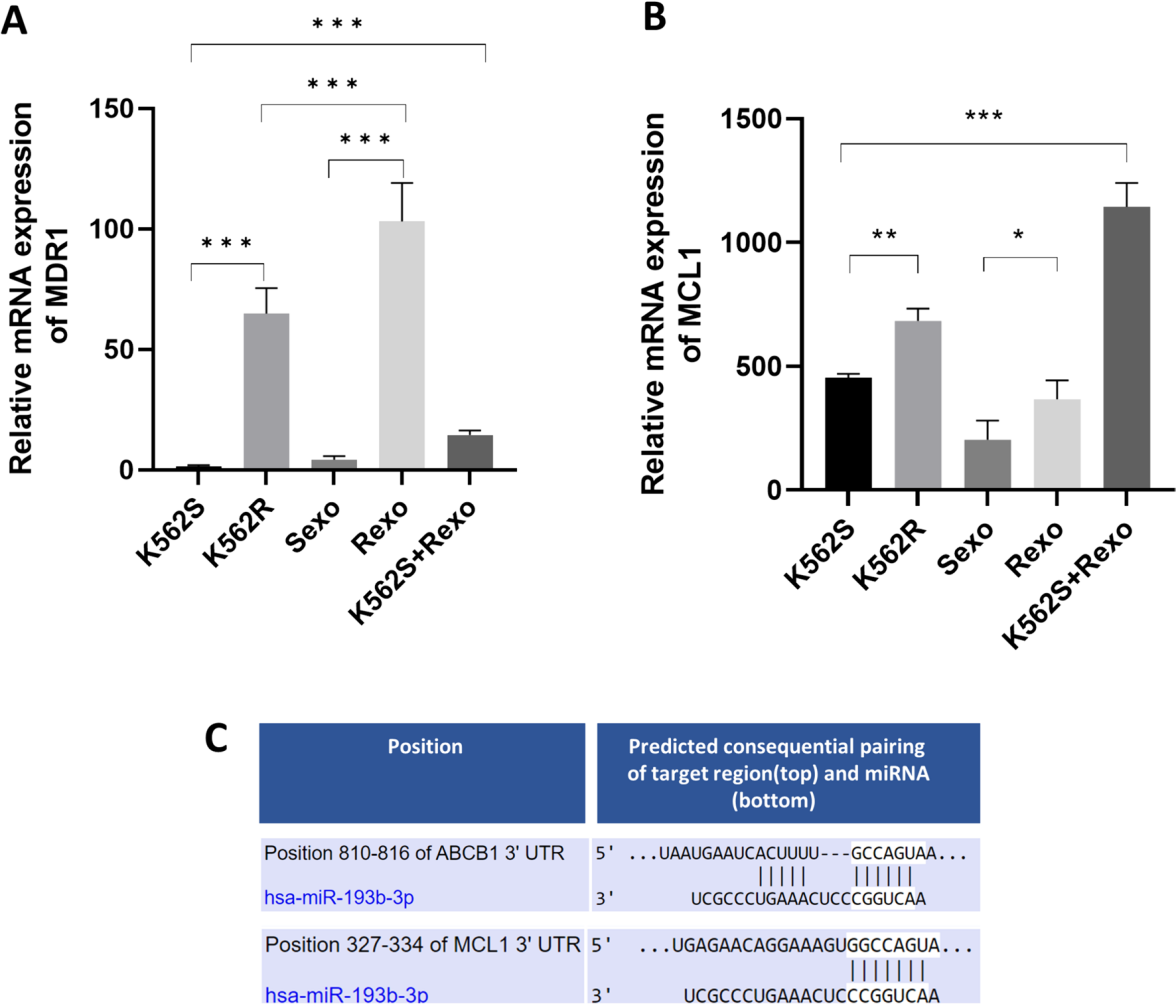
Changes in the expression of *MDR1* and *MCL1* genes and the target regions of genes for hsa-miR-193b-5p. **A** MDR1 mRNA expression in K562S, K562R, Sexo, Rexo, and K562S + Rexo. MDR1 mRNA expression was found as significantly different between Sexo vs. Rexo (*P* = 0.0004), S vs. R (*P* = 0.0005), and S vs. S + Rexo (*P* = 0.0003). **B** MCL1 mRNA expression in K562S, K562R, Sexo, Rexo, and K562S + Rexo. MDR1 mRNA expression was found as significantly different between Sexo vs. Rexo (*P* = 0.0262), S vs. R (*P* = 0.0015), and S vs. S + Rexo (*P* = 0.0003). **C** Position of genes and predicted pairing of target gene and miRNA sequences, according to TargetScan. For MCL1, TargetScan link was https://www.targetscan.org/cgi-bin/targetscan/vert_80/view_gene.cgi?rs=ENST00000369026.2&taxid=9606&members=miR-193-3p&showcnc=0&shownc=0&showncf1=&showncf2=&subset=1; for MDR1 (ABCB1), TargetScan link was: https://www.targetscan.org/cgi-bin/targetscan/vert_80/viewA_gene.cgi?rs=ENST00000265724.3&taxid=9606&members=miR-193-3p&showcnc=0&shownc=0&shownc_nc=&showncf1=&showncf2=&subset=1;bin/targetscan/vert_80/view_gene.cgi?rs = ENST00000265724.3&taxid = 9606&members = miR-193-3p&showcnc = 0&shownc = 0&shownc_nc = &showncf1 = &showncf2 = &subset = 1

The expression of the target mRNAs of miR-193b-3p was determined via RT-PCR for miRNA verification. According to TargetScan, *ABCB1* (*MDR1*) is a predicted mRNA target of miR-193b-3p. In addition, overexpression of miR-125b-5p and miR-99a-5p was reported to accompany increased *MDR1* in cisplatin resistant ovarian cancer cell lines (Kazmierczak et al. 2022). However, downregulation of miR-210-3p was reported to increase renal cell carcinoma chemotherapy resistance and high *MDR1* expression (Li et al. 2018). In addition to *MDR1*, *MCL1* was reported to be a predicted and an experimental target of miR-193b-3p by TargetScan. It was shown that the expression of *MCL1* is downregulated by overexpressed miR-193b-3p (Braconi et al. 2010; Chen et al. 2010a; Mao et al. 2014). Moreover, when miRNA-gene interaction database TarBase was searched by searching for validation of interactions between “hsa-miR-193b-3p” and “MCL1” and “hsa-miR-193b-3p” and “ABCB1 (MDR1)” using https://dianalab.e-ce.uth.gr/tarbasev9/interactions link, we found that hsa-miR-193b-3p targets and downregulates MCL1 (Chen et al. 2010a; Skalsky et al. 2012; Whisnant et al. 2013; Balakrishnan et al. 2014; Boudreau et al. 2014; Erhard et al. 2014; Yu et al. 2015; Gillen et al. 2016; Krell et al. 2016; Gay et al. 2018; Nowakowski et al. 2018; Liu and Wang 2019) and MDR1 (Boudreau et al. 2014) in multiple cell lines. The interactions provided from TarBase are also given as Supplementary data. In light of all these findings, we intended to analyze the changes in the expression of the *MDR1* and *MCL1* genes in our samples. miR-125b-5p also targets *MCL1* to suppress its activity yet we found increased *MCL1* expression which may result from the contrary effects of different miRNAs or biological components (Jia et al. 2012). Our findings revealed that the expression of both *MDR1* and *MCL1* was higher in resistant cells, in Rexo and in Rexo-treated sensitive cells than in K562S cells, Sexo, and K562S cells, respectively, which supported the findings in the literature (Fig. 10A, B). The gene expression changes we obtained are consistent with those of previously reported gene targets of miR-193b-3p. In addition, our results suggest the transport of *MDR1* to sensitive leukemia cells by exosomes from resistant leukemia cells.

### Determination of IC50 of imatinib in K562S, K562S + Rexo, and K562R cells

We characterized the phenotypic and genotypic features of the K562S and K562R cells that we used in this study in our previous studies. Our past findings revealed that factors such as suppression of apoptosis, increased autophagy, and increased *MDR1* gene expression play a role in the development of imatinib resistance, while no change was detected in Bcr-Abl gene expression (Hekmatshoar et al. 2018, 2023).

**Fig. 11:**
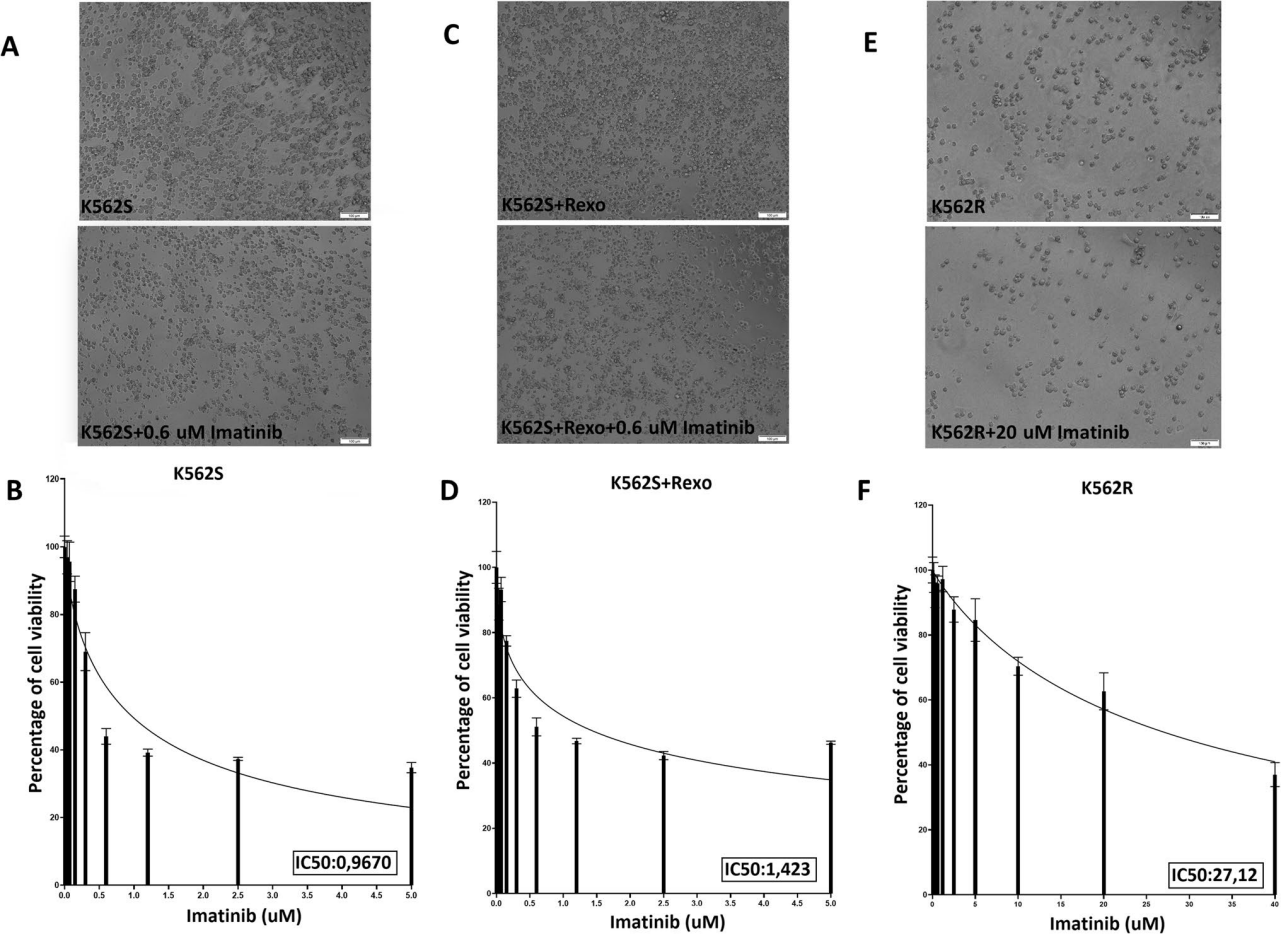
Microscopy images of K562S, K562S + Rexo and K562R cells upon imatinib exposure and IC50 graphs for imatinib. **A** Representative microscopy image of K562S cells with or without imatinib (0.6 μM). **B** Effect of different concentrations of imatinib and calculation of its IC50 in K562S cells. **C** Representative microscopy image of K562S + Rexo with or without imatinib (0.6 μM). **D** Effect of different concentrations of imatinib and calculation of its IC50 in K562S + Rexo. **E** Representative microscopy image of K562R with or without imatinib (20 μM). **F** Effect of different concentrations of imatinib and calculation of its IC50 in K562R

In the present study, we determined the IC50s of imatinib in K562S, K562S + Rexo, and K562R cells as 0.969 μM, 1.422 μM, and 27.97 μM, respectively (Fig. 11 B, D, F). Our results revealed that Rexo treatment of K562S cells increased the IC50 of imatinib. However, this increase was still much lower than the IC50 of imatinib in K562R cells. This finding may support the increase in *MDR1* gene expression, which increased with Rexo treatment but did not reach the level in resistant cells. In Fig. 11A, C, E, cell morphology can be seen in sensitive and resistant cells with or without imatinib (0.6 μM for K562S; 20 μM imatinib for K562R) treatment for 48 h.

## Discussion

In this study, we determined that a large number of miRNAs are expressed significantly different between K562R and K562S cells and between Rexo and Sexo. In a previous study, the miRNA profiles of imatinib-resistant and imatinib-sensitive K562 cell-derived exosomes were compared, and the miRNA with the most significant fold change was determined to be miR-365 which was also shown to be transferred to sensitive cells via exosomes (Min et al. 2018). In the same study, 6 miRNAs that showed significant decreases in resistant cells compared to sensitive cells exhibited similar expression changes in our study. On the other hand, one of the commonly found miRNAs, and miR-629-5p exhibited decreased expression in Rexo in our study contrary to the findings of this study (Min et al. 2018). Apart from these miRNAs, no common miRNAs were found in our study.

According to our data, intersecting differentially expressed miRNAs between K562S and K562R cells, their exosomes and K562S cells and K562S cells treated with Rexo revealed 28 commonly expressed miRNAs. Among these miRNAs, miR-99a-5p and miR-125b-5p exhibited increased expression in resistant profile. On the other hand, miR-210-3p and miR-193b-3p were found as the only two miRNAs with decreased expression in resistant profiles among these 28 miRNAs. Three of these miRNAs have been reported in the literature for their association with leukemogenesis and therapy response. However, their role in exosomal transfer of resistance profile has not been reported before, and we reveal their involvement in exosomal resistance transfer for the first time.

In the literature, increased expression of miR-99a-5p was reported in nonmuscle invasive bladder cancer (Kaba et al. 2023), cisplatin-resistant esophageal cancer cells (Pandey et al. 2023), topotecan resistant ovarian cancer cell lines (Stasiak et al. 2022), cisplatin resistant ovarian cancer cell lines (Kazmierczak et al. 2022), diffuse large B-cell lymphoma with chemotherapy resistance (Feng et al. 2019), and signet-ring cell carcinoma, a histological subtype of gastric cancer (Saito et al. 2020). In parallel with these studies, it has been shown that exosomal miR-99a-5p is elevated in the serum of ovarian cancer patients and promotes cancer cell invasion by increasing fibronectin and vitronectin expression in adjacent peritoneal mesothelial cells (Yoshimura et al. 2018). On the contrary, some studies have reported decreased expression of this miRNA in cancer. Its expression has been shown to decrease in head and neck squamous cell carcinoma (Chen et al. 2018), doxorubicin-resistant breast cancer cells (Garrido-Cano et al. 2022), oral squamous cell carcinoma (Sun and Yan 2021), and breast cancer tissues (Garrido-Cano et al. 2020). Its overexpression has been found to reduce human oral carcinoma cell proliferation (Shi et al. 2017), and breast cancer progression (Qin and Liu 2019) and its downregulation were reported to mediate bladder cancer formation (Liu et al. 2019b). All these findings reveal mixed functions for miR-99a-5p some of which reveal tumor suppressor activity whereas others indicate oncogenic functions. This discrepancy may stemmed from different sample sources of miRNAs or distinct cancer types.

In leukemia, miR-99a upregulation was reported to be associated with stemness of acute myeloid leukemia cells and to be correlated with leukemia progression and worse overall survival. It was also reported that miR-99a was upregulated in imatinib-resistant K562 cells and that its ectopic expression induced leukemic cell survival after exposure to chemotherapeutic agents (Si et al. 2016). Our findings of high miR-99a-5p expression in drug-resistant K562 cells and their exosomes support and contribute to these findings by demonstrating the exosomal transfer of miR-99a in imatinib resistance of CML for the first time.

The other highly expressed miRNA that we detected in resistant cells and their exosomes was miR-125b-5p. Both tumor promoting and tumor suppressing activities of miR-125b-5p have been reported in the literature. miR-125b-5p was reported to increase in different cancers including breast cancer (Incoronato et al. 2019), lung adenocarcinoma (Zeybek et al. 2019), multiple myeloma (Jiang et al. 2018), cisplatin and sorafenib-resistant cancers (Hirao et al. 2021; Tan et al. 2022; Roška et al. 2023), and diffuse large B-cell lymphoma (Feng et al. 2019). Its overexpression was shown to be associated with inflammation induced colon cancer (Abramczyk et al. 2023) whereas its exosomal delivery from cancer associated fibroblasts was reported to induce the metastasis and invasion of pancreatic cancer (Guo, Li and Sun, 2023). In addition, silencing miR-125b-5p was reported to inhibit the malignant behaviors of nasopharyngeal carcinoma cells (Peng et al. 2020). On the other hand, in a number of other studies, miR-125b-5p was linked to tumor suppressor activity. Its upregulation was reported to be linked to decreased ovarian tumor growth (Liu et al. 2019a), reversal of colon cancer drug resistance (ParkJeong and Kim, 2020, Shi et al. 2020), and increased tamoxifen sensitivity of breast cancer cells (Li et al. 2019) and suppression of growth in different types of cancers (Guo et al. 2019, Liu Chen and Wang, 2020, Huang et al. 2022, Pan et al. 2023, Tan et al. 2023).

Most importantly, when we checked the literature findings for the involvement of miR-125b in leukemia, miR-125b overexpression was reported to accelerate the oncogenic nature of the BCR-ABL oncoprotein as a trigger to induce primary lymphoid or myeloid leukemia and was therefore to be considered a therapy target (Bousquet et al. 2010). In parallel with the findings of this study and with the literature findings which associate oncogenic activity and chemotherapy resistance with miR-125b-5p, we found increased expression of this miRNA in resistant cells and their exosomes which may support possible exosomal transportation to mediate imatinib resistance.

Considering the groups we compared, two miRNAs, miR-210-3p and miR-193b-3p, exhibited decreased expression in the resistance profile. In the literature, increased miR-210-3p expression was reported in pancreatic cancer (Qu et al. 2017), small-cell lung cancer (Han et al. 2020), and breast cancer (Gong et al. 2020) whereas miR-210-3p was postulated to be a hypoxia induced miRNA in breast cancer, lung cancer, and glioblastoma (Gong et al. 2020; Liu et al. 2021; Arora et al. 2022). Oncogenic activity has been reported for this miRNA in the progression of lung and endometrial carcinoma (Han et al. 2020; Dai et al. 2022). On the other hand, in a study, it was reported that the desired pathological response was achieved with high expression of miR-210-3p in breast cancer (Lisencu et al. 2022). These studies collectively reported the different activities of miR-210-3p in different cancer types. In contrast to our findings of decreased exosomal expression of miR-210-3p in imatinib resistance, exosomal miR-210-3p was shown to be upregulated in osimertinib-resistant non-small-cell lung cancer cell lines (Hisakane et al. 2021).

In addition to miR-210-3p, we detected decreased expression of miR-193b-3p in resistant cells, their exosomes, and sensitive cells treated with Rexo. Our findings support the literature data which reports 4.4-fold significant downregulation of miR-193b-5p expression in imatinib-resistant CML patients compared to patients who respond to imatinib (Ramachandran et al. 2017). Various studies have reported the tumor suppressor activity of miR-193b-3p in different cancer types including melanoma, breast cancer, epidermal squamous cell carcinoma, lung cancer, and gastric cancer (Li Yan and Shao, 2009, Chen et al. 2010a; Gastaldi et al. 2014; Song et al. 2018; Choi et al. 2019). On the other hand, differently from our findings pointing out decreased expression in resistance, miR-193b-3p was reported to be enriched in exosomes of cisplatin resistant seminoma cells (Wang et al. 2022).

Another striking finding we determined was higher *MDR1* and *MCL1* gene expression in exosomes derived from imatinib resistant cells as well as Rexo-treated K562S cells compared to K562S cells, revealing that *MDR1* is an important mediator of drug resistance and is transferred from sensitive cells to resistant cells. Our results support the findings of previous reports of in vitro exosomal transfer of *MDR1/P-gp* mRNA and protein from doxorubicin resistant human osteosarcoma (Torreggiani et al. 2016), docetaxel resistant prostate cancer (Corcoran et al. 2012), and breast cancer (Lv et al. 2014) cells to their sensitive counterparts as well as in vivo data showing intercellular transfer of P-gp in tumor cells (Levchenko et al. 2005).

In the literature, the role of MDR1 in the response to imatinib in CML patients has been investigated in various studies. In one of the studies, lower *MDR1* mRNA levels were reported in patients who showed an optimal response to the drug compared to patients who did not show an optimal response and showed resistance (Bedewy et al. 2019). In another study, patients with a high fold increase in *MDR1* gene expression were found to be less prone to early and major molecular response, and it was suggested that fold changes in *MDR1* mRNA could be used to determine imatinib response. Additionally, progression to blast crisis and the emergence of mutations have been reported in patients exhibiting high *MDR1* (Eadie et al. 2017). Accelerated phase (AP) and blastic crisis (BC) CML patients were reported to express higher *MDR1* expression compared to chronic phase (CP) CML patients supporting these findings (Solali et al. 2013). In another study, secondary imatinib resistance was linked with high *MDR1* expression (Zhang et al. 2009). On the other hand, it has been reported that 6 and 12 months of imatinib treatment did not create a significant difference in *MDR1* levels in different cell populations of CML patients between pre-treatment and post-treatment. Researchers stated that the observation time and number of patients in their study were limited, but they still reported that a significant number of patients were able to respond optimally despite having high *MDR1* levels (Razga et al. 2011). Similarly, in another study, *MDR1* mRNA expression was not shown to be correlated with molecular response in CML patients. However, it was stated that the findings obtained in this study should be confirmed with a larger number of patients (Malhotra et al. 2015).

In the literature, multiple studies on cell lines have shown that continuous imatinib exposure leads to an increase in *MDR1* expression and imatinib resistance (Tang et al. 2011, Kosztyu et al. 2014, Eadie et al. 2016, Hekmatshoar et al. 2018).

Besides MDR1, MCL1 was extensively studied in cancer and overexpression, and amplification of *MCL1*, a Bcl-2 family member anti-apoptotic protein, has been linked with poor prognosis in various solid (Maroufi et al. 2020) and hematological cancers including AML and lymphoma (Wei et al. 2020; Wang et al. 2021). In a study, it was reported that *MCL1* was expressed in all CML patient samples studied, regardless of the phase of CML disease, and that immune-reactive MCL1 exhibited higher expression in bone marrow mononuclear cells of CML patients than in normal bone marrow. In the same study, the CML cell line K562 was also used, and it was reported that MCL1 blockade increased the imatinib response in both sensitive and resistant cells (Aichberger et al. 2005).

In parallel with these results, in a study using cell lines and CML samples, the combined treatment of imatinib and S63845, a MCL1 inhibitor, was reported to exhibit apoptotic effects on primary human CD34 + CML stem/progenitor cells and sensitive and resistant cell lines. Since these stem/progenitor cells are mainly responsible for the emergence of therapy resistance after long-term tyrosine kinase inhibitor treatment, combinational therapy of MCL1 inhibition and TKIs may aid in reversal of resistance (Malyukova et al. 2021). On the other hand, in another study conducted on chronic phase CML patients, no correlation was found between *MCL1* level and assessment of prognosis, but researchers suggested that the role of MCL1 in the development of drug resistance should be further examined with other studies using more samples (Darojat et al. 2023).

Collectively, data from various clinical and cell line studies suggest that *MDR1* and *MCL1* may play an active role in chemotherapy response and imatinib resistance and our findings may suggest the involvement of these genes in exosomal resistance transfer.

In addition, we used various bioinformatic tools to analyze the interacting genes and protein interaction networks of the discovered miRNAs. STRING analysis of the PPI network and determination of target hub genes using Cytoscape revealed ten genes with the highest ranking scores for each set of miRNAs with increased or decreased expression. Among these genes, *MTOR* (Burchert et al. 2005), *STAT3*, *MCL1* (Bewry et al. 2008), *LAMC1* (Zhang et al. 2022), and *KRAS* (Agarwal et al. 2007) have been reported to play roles in imatinib resistance in chronic myeloid leukemia in parallel with our findings. Other genes such as *CCR5*, *GRK2*, *EDN1*, *ARRB1*, *P2RY2*, *PPP2CA*, *PPP2R5C*, *LAMC2*, *PAK3*, *PAK4*, *LAMC1*, *GIT2*, *PIK3R1*, *UBA52*, *NCOR1*, and *BDNF* may be revealed as novel genes related to imatinib resistance. We also found that these genes are involved in the PI3K-Akt signaling pathway, microRNAs in cancer, Ras signaling pathway, and cancer pathways which collectively take part in the progression of cancer and development of chemotherapy resistance.

Taken together, our results reveal that the transfer of miRNAs and *MDR1* via exosomes may serve as a mechanism of imatinib resistance development in chronic myeloid leukemia. miR-210-3p, miR-193b-3p, miR-125b-5p, and miR-99a-5p may be involved in the resistance processes by targeting different signaling events and genes, and targeting these miRNAs may be examined as a therapeutic strategy to overcome imatinib resistance.

## Supplementary Information

Below is the link to the electronic supplementary material.

Supplementary file1 (DOCX 73 KB)

## Data Availability

Data supporting the findings of this study are available to readers upon reasonable request.
