# Supplementary material for: Identification of exosomal microRNAs and related hub genes associated with imatinib resistance in chronic myeloid leukemia
Source: Naunyn Schmiedebergs Arch Pharmacol. 2024 Jun 25;397(12):9701–21. doi: 10.1007/s00210-024-03198-1 (PMC11582232; doi:10.1007/s00210-024-03198-1)
Supplement: Supplementary file 1 — Supplementary file1 (DOCX 73 KB) [file 210_2024_3198_MOESM1_ESM.docx]

| **Gene name** | **miRNA name** | **Experiments** | **Publications** | **Cell lines** |
| --- | --- | --- | --- | --- |
| MCL1 | hsa-miR-193b-3p | 14 | 11 | 10 |

| **Publication** | **Tissue** | **Cell line** | **Cell type** | **Treatment** | **Experimental method** | **Experimental type** | **Phenotype** |
| --- | --- | --- | --- | --- | --- | --- | --- |
| [*Chen J et al. et al. 2010*](https://pubmed.ncbi.nlm.nih.gov/20304954) | NA | NA | NA | NA | Microarrays | Indirect | Cancer/Malignant |
| \| **Binding site** \| **Transcript name** \| **Transcript id** \| **Transcript location** \| **Regulation** \| **Validation Type** \| \| --- \| --- \| --- \| --- \| --- \| --- \| \| NA \| MCL1-202 \| [ENST00000369026](https://www.ensembl.org/Homo_sapiens/Transcript/Summary?db=core;t=ENST00000369026) \| NA \| Down \| Positive \| | | | | | | | |
| [*Skalsky RL et al et al. 2012*](https://pubmed.ncbi.nlm.nih.gov/22291592) | NA | EF3DAGO2 | B lymphocytes | EBV-infected cells | PAR-CLIP | Direct | Normal/Primary |
| \| **Binding site** \| **Transcript name** \| **Transcript id** \| **Transcript location** \| **Regulation** \| **Validation Type** \| \| --- \| --- \| --- \| --- \| --- \| --- \| \| chr1 \| - \| 150576053-150576065 \| NA \| [ENST00000369026](https://www.ensembl.org/Homo_sapiens/Transcript/Summary?db=core;t=ENST00000369026) \| 3UTR \| Down \| Positive \| \| chr1 \| - \| 150576960-150576972 \| NA \| [ENST00000369026](https://www.ensembl.org/Homo_sapiens/Transcript/Summary?db=core;t=ENST00000369026) \| 3UTR \| Down \| Positive \| \| chr1 \| - \| 150578259-150578271 \| NA \| [ENST00000369026](https://www.ensembl.org/Homo_sapiens/Transcript/Summary?db=core;t=ENST00000369026) \| CDS \| Down \| Positive \| | | | | | | | |
| [*Whisnant AW et al et al. 2013*](https://pubmed.ncbi.nlm.nih.gov/23592263) | Cervix | TZMBL | CD4+ T cells | HIV-1-infected cells | PAR-CLIP | Direct | Cancer/Malignant |
| \| **Binding site** \| **Transcript name** \| **Transcript id** \| **Transcript location** \| **Regulation** \| **Validation Type** \| \| --- \| --- \| --- \| --- \| --- \| --- \| \| chr1 \| - \| 150578259-150578271 \| NA \| [ENST00000369026](https://www.ensembl.org/Homo_sapiens/Transcript/Summary?db=core;t=ENST00000369026) \| CDS \| Down \| Positive \| | | | | | | | |
| [*Erhard F et al. 2014*](https://pubmed.ncbi.nlm.nih.gov/24668909) | Bone marrow | BCBL-1 | B lymphocytes | KSHV-infected cells | PAR-CLIP | Direct | Cancer/Malignant |
| \| **Binding site** \| **Transcript name** \| **Transcript id** \| **Transcript location** \| **Regulation** \| **Validation Type** \| \| --- \| --- \| --- \| --- \| --- \| --- \| \| chr1 \| - \| 150578259-150578271 \| NA \| [ENST00000369026](https://www.ensembl.org/Homo_sapiens/Transcript/Summary?db=core;t=ENST00000369026) \| CDS \| Down \| Positive \| | | | | | | | |
| [*Ilango Balakrishnan et al. 2014*](https://pubmed.ncbi.nlm.nih.gov/24038734) | Bone marrow | HS-5 | Mesenchymal stem cells | Untreated | HITS-CLIP | Direct | Normal/Primary |
| \| **Binding site** \| **Transcript name** \| **Transcript id** \| **Transcript location** \| **Regulation** \| **Validation Type** \| \| --- \| --- \| --- \| --- \| --- \| --- \| \| chr1 \| - \| 150578259-150578271 \| MCL1-208 \| [ENST00000678770](https://www.ensembl.org/Homo_sapiens/Transcript/Summary?db=core;t=ENST00000678770) \| CDS \| Down \| Positive \| | | | | | | | |
| [*Feng Yu et al. 2015*](https://pubmed.ncbi.nlm.nih.gov/26061048) | Breast | MDA-MB-231 | Epithelial cells | Transfected with hsa-miR-200a mimic | HITS-CLIP | Direct | Cancer/Malignant |
| \| **Binding site** \| **Transcript name** \| **Transcript id** \| **Transcript location** \| **Regulation** \| **Validation Type** \| \| --- \| --- \| --- \| --- \| --- \| --- \| \| chr1 \| - \| 150578259-150578271 \| MCL1-208 \| [ENST00000678770](https://www.ensembl.org/Homo_sapiens/Transcript/Summary?db=core;t=ENST00000678770) \| CDS \| Down \| Positive \| | | | | | | | |
| [*Austin E Gillen et al. 2016*](https://pubmed.ncbi.nlm.nih.gov/27150721) | Breast | MCF-7 | Epithelial cells | 10nM 17b-estradiol for 24 hours | HITS-CLIP | Direct | Cancer/Malignant |
| \| **Binding site** \| **Transcript name** \| **Transcript id** \| **Transcript location** \| **Regulation** \| **Validation Type** \| \| --- \| --- \| --- \| --- \| --- \| --- \| \| chr1 \| - \| 150578259-150578271 \| MCL1-208 \| [ENST00000678770](https://www.ensembl.org/Homo_sapiens/Transcript/Summary?db=core;t=ENST00000678770) \| CDS \| Down \| Positive \| | | | | | | | |
| [*Austin E Gillen et al. 2016*](https://pubmed.ncbi.nlm.nih.gov/27150721) | Breast | MCF-7 | Epithelial cells | 10nM 17b-estradiol for 6 hours | HITS-CLIP | Direct | Cancer/Malignant |
| \| **Binding site** \| **Transcript name** \| **Transcript id** \| **Transcript location** \| **Regulation** \| **Validation Type** \| \| --- \| --- \| --- \| --- \| --- \| --- \| \| chr1 \| - \| 150578259-150578271 \| MCL1-208 \| [ENST00000678770](https://www.ensembl.org/Homo_sapiens/Transcript/Summary?db=core;t=ENST00000678770) \| CDS \| Down \| Positive \| | | | | | | | |
| [*Krell J et al. 2016*](https://pubmed.ncbi.nlm.nih.gov/26701625) | Intestine | HCT116 | Epithelial cells | Untreated | PAR-CLIP | Direct | Cancer/Malignant |
| \| **Binding site** \| **Transcript name** \| **Transcript id** \| **Transcript location** \| **Regulation** \| **Validation Type** \| \| --- \| --- \| --- \| --- \| --- \| --- \| \| chr1 \| - \| 150578259-150578271 \| NA \| [ENST00000369026](https://www.ensembl.org/Homo_sapiens/Transcript/Summary?db=core;t=ENST00000369026) \| CDS \| Down \| Positive \| | | | | | | | |
| [*Gay LA et al. 2018*](https://pubmed.ncbi.nlm.nih.gov/29386283) | Umbilical vein | TIVE-LTC | Endothelial cells | KSHV-infected (lacking kshv-miR-K12-11) | qCLASH | Direct | Telomerase-immortalized |
| \| **Binding site** \| **Transcript name** \| **Transcript id** \| **Transcript location** \| **Regulation** \| **Validation Type** \| \| --- \| --- \| --- \| --- \| --- \| --- \| \| chr1 \| - \| 150578572-150578652 \| MCL1-208 \| [ENST00000678770](https://www.ensembl.org/Homo_sapiens/Transcript/Summary?db=core;t=ENST00000678770) \| CDS \| Down \| Positive \| \| chr1 \| - \| 150578594-150578644 \| MCL1-208 \| [ENST00000678770](https://www.ensembl.org/Homo_sapiens/Transcript/Summary?db=core;t=ENST00000678770) \| CDS \| Down \| Positive \| \| chr1 \| - \| 150578596-150578652 \| MCL1-208 \| [ENST00000678770](https://www.ensembl.org/Homo_sapiens/Transcript/Summary?db=core;t=ENST00000678770) \| CDS \| Down \| Positive \| | | | | | | | |
| [*Gay LA et al. 2018*](https://pubmed.ncbi.nlm.nih.gov/29386283) | Umbilical vein | TIVE-LTC | Endothelial cells | KSHV-infected (wild-type) | qCLASH | Direct | Telomerase-immortalized |
| \| **Binding site** \| **Transcript name** \| **Transcript id** \| **Transcript location** \| **Regulation** \| **Validation Type** \| \| --- \| --- \| --- \| --- \| --- \| --- \| \| chr1 \| - \| 150578599-150578635 \| MCL1-208 \| [ENST00000678770](https://www.ensembl.org/Homo_sapiens/Transcript/Summary?db=core;t=ENST00000678770) \| CDS \| Down \| Positive \| | | | | | | | |
| [*Gay LA et al. 2018*](https://pubmed.ncbi.nlm.nih.gov/29386283) | Umbilical vein | TIVE-LTC | Endothelial cells | Untreated | qCLASH | Direct | Telomerase-immortalized |
| \| **Binding site** \| **Transcript name** \| **Transcript id** \| **Transcript location** \| **Regulation** \| **Validation Type** \| \| --- \| --- \| --- \| --- \| --- \| --- \| \| chr1 \| - \| 150578581-150578631 \| MCL1-208 \| [ENST00000678770](https://www.ensembl.org/Homo_sapiens/Transcript/Summary?db=core;t=ENST00000678770) \| CDS \| Down \| Positive \| | | | | | | | |
| [*Tomasz J Nowakowski et al. 2018*](https://pubmed.ncbi.nlm.nih.gov/30455455) | Brain - Primary Visual Cortex | NA | NA | Untreated | HITS-CLIP | Direct | Normal/Primary |
| \| **Binding site** \| **Transcript name** \| **Transcript id** \| **Transcript location** \| **Regulation** \| **Validation Type** \| \| --- \| --- \| --- \| --- \| --- \| --- \| \| chr1 \| - \| 150578259-150578271 \| MCL1-208 \| [ENST00000678770](https://www.ensembl.org/Homo_sapiens/Transcript/Summary?db=core;t=ENST00000678770) \| CDS \| Down \| Positive \| | | | | | | | |
| [*Weijun Liu et al. 2019*](https://pubmed.ncbi.nlm.nih.gov/30670076) | Cervix | HeLa | Epithelial cells | Overexpression of hsa-miR-193b-3p | RNA-Seq | Indirect | Cancer cell line |
| \| **Binding site** \| **Transcript name** \| **Transcript id** \| **Transcript location** \| **Regulation** \| **Validation Type** \| \| --- \| --- \| --- \| --- \| --- \| --- \| \| NA \| MCL1-208 \| [ENST00000678770](https://www.ensembl.org/Homo_sapiens/Transcript/Summary?db=core;t=ENST00000678770) \| NA \| Down \| Positive \| | | | | | | | |

| **Gene name** | **miRNA name** | **Experiments** | **Publications** | **Cell lines** | | **microT Score** |
| --- | --- | --- | --- | --- | --- | --- |
| ABCB1 | hsa-miR-193b-3p | 1 | 1 | | 1 | [0.40](https://dianalab.e-ce.uth.gr/microt_webserver/#/interactions?mirnas=hsa-miR-193b-3p&genes=ENSG00000085563) |

| **Publication** | **Tissue** | **Cell line** | **Cell type** | **Treatment** | **Experimental method** | **Experimental type** | **Phenotype** |
| --- | --- | --- | --- | --- | --- | --- | --- |
| [*Ryan L Boudreau et al. 2014*](https://pubmed.ncbi.nlm.nih.gov/24389009) | Brain - Cingulate gyrus | NA | NA | Untreated | HITS-CLIP | Direct | Post-mortem |
| \| **Binding site** \| **Transcript name** \| **Transcript id** \| **Transcript location** \| **Regulation** \| **Validation Type** \| \| --- \| --- \| --- \| --- \| --- \| --- \| \| chr7 \| - \| 87550048-87550060 \| ABCB1-201 \| [ENST00000265724](https://www.ensembl.org/Homo_sapiens/Transcript/Summary?db=core;t=ENST00000265724) \| CDS \| Down \| Positive \| | | | | | | | |
